# Supplementary material for: Coronin 1C, Regulated by Multiple microRNAs, Facilitates Cancer Cell Aggressiveness in Pancreatic Ductal Adenocarcinoma
Source: Genes (Basel). 2023 Apr 27;14(5):995. doi: 10.3390/genes14050995 (PMC10218124; doi:10.3390/genes14050995)
Supplement: Supplementary file 1 [file genes-14-00995-s001.zip › Supplementary Figures.pptx]

## Slide 1
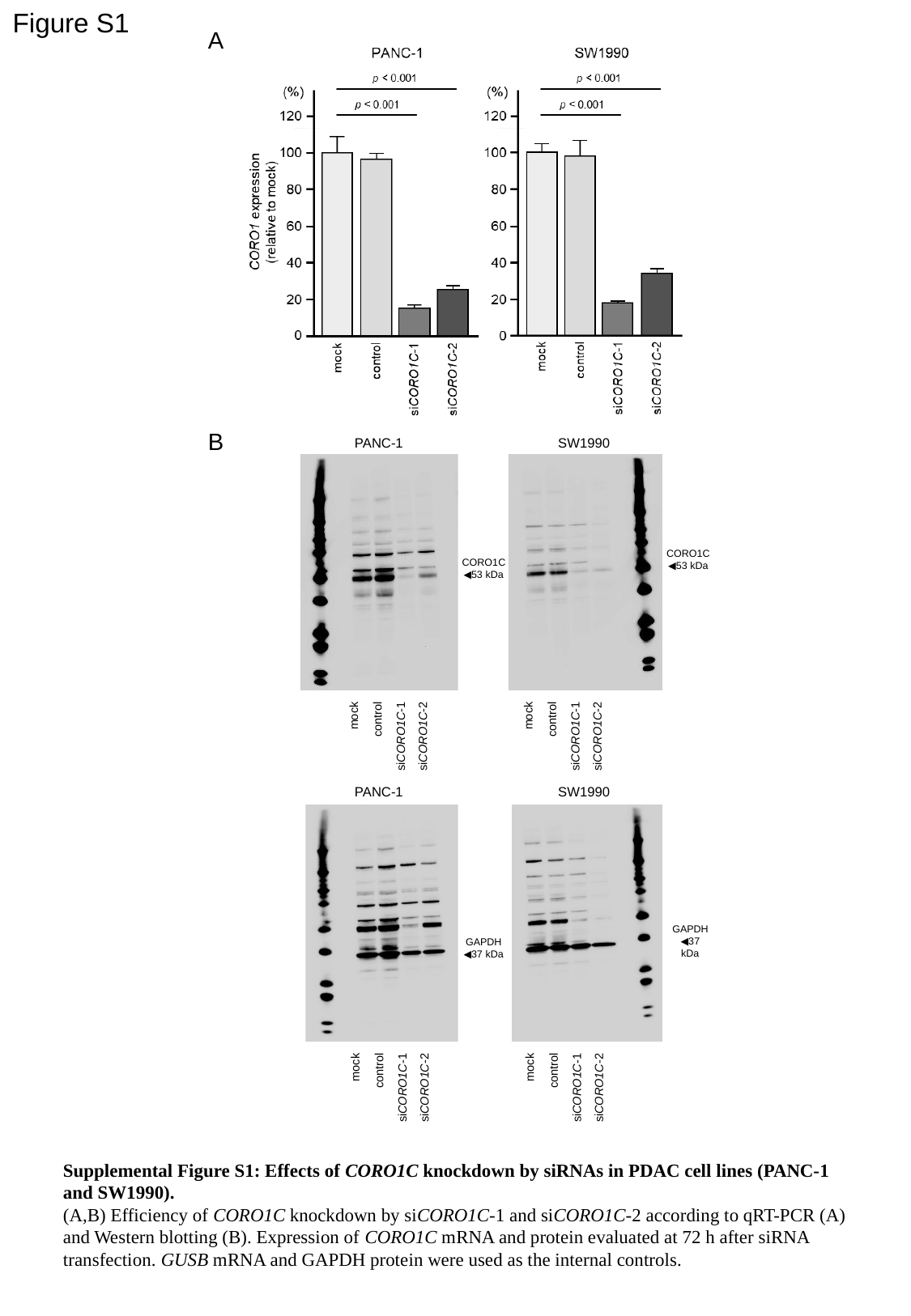

Figure S1
A
B
PANC-1
SW1990
CORO1C
◀53 kDa
CORO1C
◀53 kDa
mock
control
siCORO1C-1
siCORO1C-2
mock
control
siCORO1C-1
siCORO1C-2
PANC-1
SW1990
GAPDH
◀37 kDa
GAPDH
◀37 kDa
mock
control
siCORO1C-1
siCORO1C-2
mock
control
siCORO1C-1
siCORO1C-2
Supplemental Figure S1: Effects of CORO1C knockdown by siRNAs in PDAC cell lines (PANC-1 and SW1990).
(A,B) Efficiency of CORO1C knockdown by siCORO1C-1 and siCORO1C-2 according to qRT-PCR (A) and Western blotting (B). Expression of CORO1C mRNA and protein evaluated at 72 h after siRNA transfection. GUSB mRNA and GAPDH protein were used as the internal controls.

## Slide 2
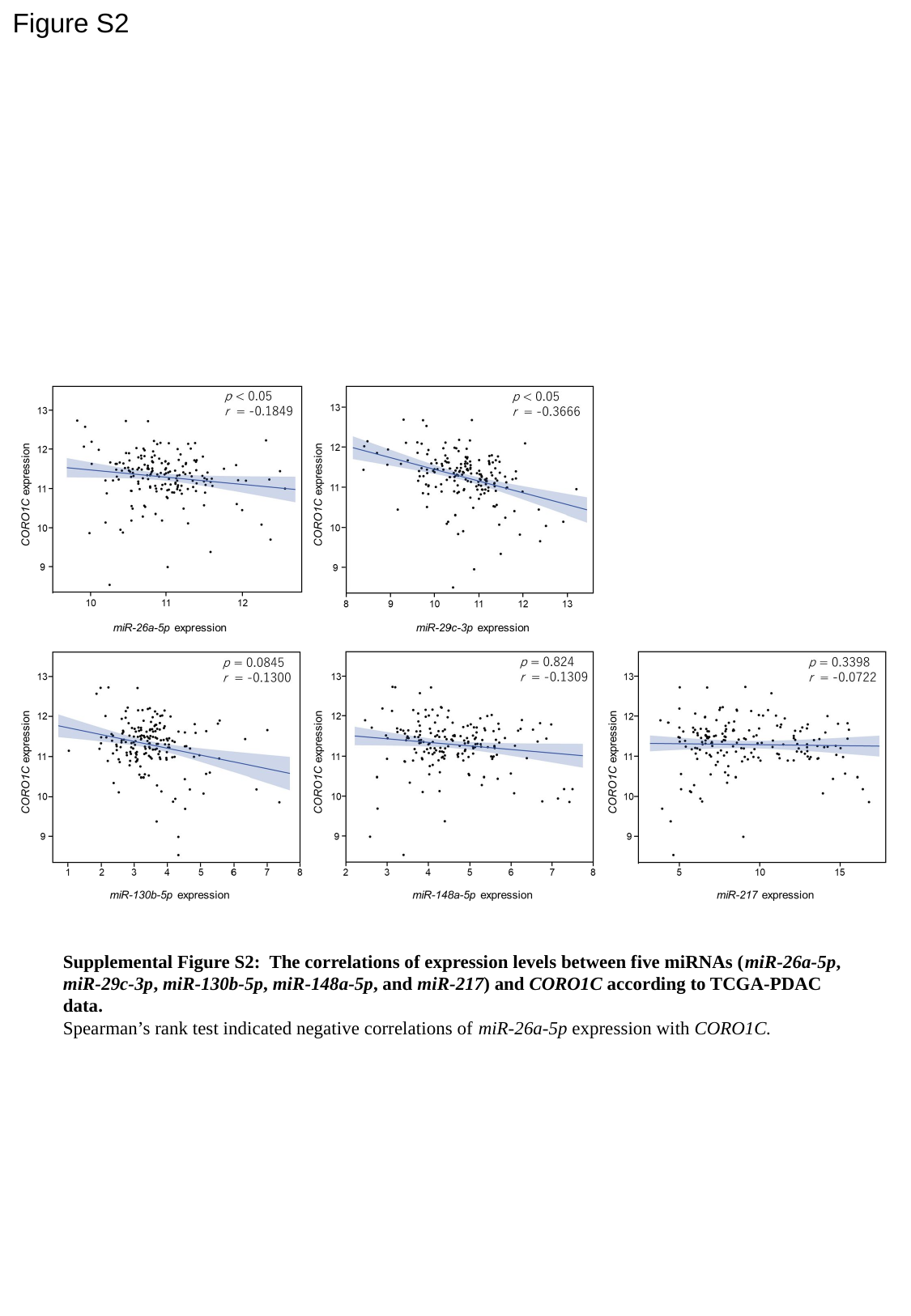

Figure S2
Supplemental Figure S2: The correlations of expression levels between five miRNAs (miR-26a-5p, miR-29c-3p, miR-130b-5p, miR-148a-5p, and miR-217) and CORO1C according to TCGA-PDAC data.
Spearman’s rank test indicated negative correlations of miR-26a-5p expression with CORO1C.

## Slide 3
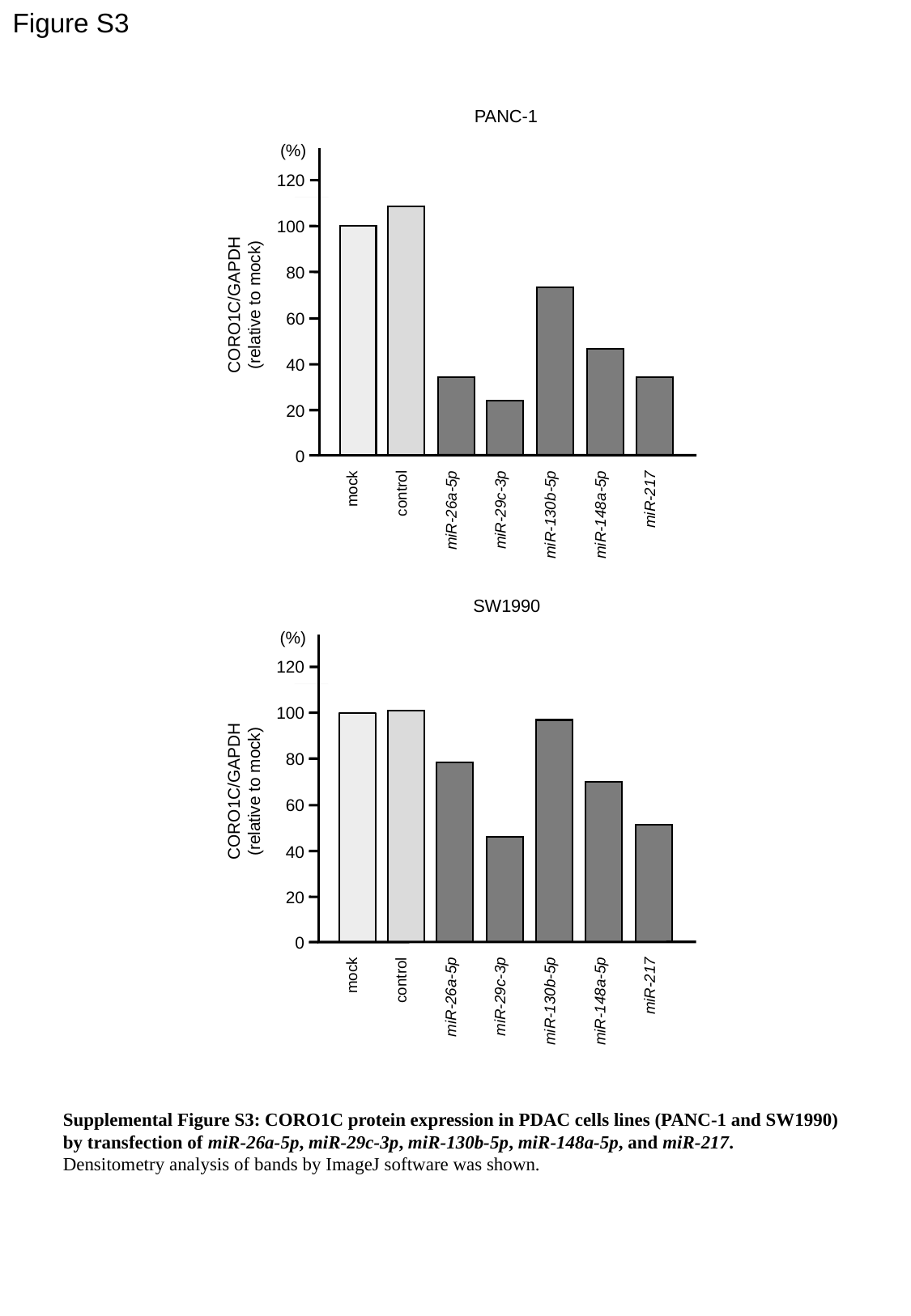

Figure S3
PANC-1
(%)
CORO1C/GAPDH
(relative to mock)
120
100
80
60
40
20
0
mock
control
miR-26a-5p
miR-29c-3p
miR-130b-5p
miR-148a-5p
miR-217
SW1990
(%)
CORO1C/GAPDH
(relative to mock)
120
100
80
60
40
20
0
mock
control
miR-26a-5p
miR-29c-3p
miR-130b-5p
miR-148a-5p
miR-217
Supplemental Figure S3: CORO1C protein expression in PDAC cells lines (PANC-1 and SW1990) by transfection of miR-26a-5p, miR-29c-3p, miR-130b-5p, miR-148a-5p, and miR-217.
Densitometry analysis of bands by ImageJ software was shown.
